# Report of Deaths in the City of Buffalo, for the Month of August, 1864

**Published:** 1864-11

**Authors:** 


*Report of Deaths in the City of Buffalo, for the month of August*, 1864.

Sex.—Males, 103 ; Females, 104. Total 207.

Nativities.—United States 162, German States 18, Ireland 16, England 1, France 1, Scotland 2, Canada 4, Denmark 1, Italy 1, Sweden 1.

Parentage.—American 42, German 103, Irish 40, English 7, Scotch 5, French 1, Denmark I, Prussia 1, Canada 3, Italy 1, Sweden 1, unknown 2. Total 207.

Condition.—Married 38, single 162, widows 3, widowers 4, unknown 10.

Locality.—City at large 182, Hospital of Sisters of Charity 10, Buffalo General Hospital 3. Catholic Foundling Asylum 5, Erie County Alms House 7, Total 207.

Ages.—1 day to 30 days 9, 1 month to 6 months 21, 6 months to 1 year 41, 1 to 3 years 49, 8 to 5 years 6. 5 to 10 years 11, 10 to 20 years 15, 20 to 30 years 11, 30 to 40 years 5, 40 to 45 years 14, 50 to 60 years 8, 60 to 70 years 8, 70 to 80 years 3, 80 to 90 years 2, 90 to 100 years 1, stillborn 2, unknown 1. Total 207.

By whom Certified.—By Regular Physicians at Public Institutions 25, by Regular Physicians in city at large 96, by Irregular Practitioners 44, by Coroner 8, by Undertakers 34. Total 207.

CAUSES OF DEATH.—Accident 3, do. by burn 1, do. by drowning 3, anemia 2, apoplezy, cerebral 2, brain, compression of 1, bronchitis 1, cancer of womb 1, cholara Asiatic 1, do. infantum 34, consumption 12, convulsions 5, croup 3, croup diphtheritic 1, debility 3, dentition 3, diarrhœa 47, disease of the brain 2, do. heart 3, do. stomach 3, diphtheria 4, dysentery 17, fever 2, do. scarlet 5, do. typhoid 4, gangrene (hosp.) 1, hemorrhage from lungs 1, inflammation of bladder 1, do. bowels 2, do. brain 2, do. brain and meninges 4, do. liver 1, do. lungs 2, do. lungs typhoid 1, intemperance 1, jaundice 1, mania puerperal 1, marasmus 1, measles 5, old age 3. premature birth 1. pyæmia 2, perforation of bowels 1, scrofula 1. suicide, (poison) 1, unknown 4. Deaths from diseases 205.

The number of deaths in the first eight months of the present year, is 58 *more* than in the corresponding period of l6st year; and 56 *more* than the average for five years.

SANDFORD B. EASTMAN, M. D.,
Health Physician.

### *Report of Deaths in the City of Buffalo, for the month of September,* 1864

SEX.—Males, 86; Females, 59; Total, 145.

NATIVITIES.—United States, 111; German States, 11; Ireland, 13; England 2; France, 1; Scotland, 1; Prussia, 1; Unknown, 5.

PARENTAGE.—American, 34; German, 62; Irish, 30; English, 8; Scotch, 1; French, 3; Prussia, 1; Italy, 1; Unknown, 5.

CONDITION.—Married, 23; Single, 105; Widows, 9, Widowers, 2; Unknown, 6.

LOCALITY.—City at large, 129; Hospital of Sisters of Charity, 6; Buffalo General Hospital, 2; Erie County Alms House, 6; Providence Insane Asylumn, 1; Soldiers' Rest, 1.

AGES.—1 day to 30 days, 7; 1 month to 6 months, 5; 6 months to 1 year, 17; 1, to 3 years, 34; 3 to 5 years, 12; 5 to 10 years 8; 10 to 20 years. 7; 20 to 30 years, 11; 30 to 40 years, 8; 40 to 50 years, 10; 50 to 60 years, 7; 60 to 70 years, 7; 70 to 80 years, 7; Stillborn, 4; Unknown, 1.

BY WHOM CERTIFIED.—By Regular Physicians at Public In stitutions, 14; by Regular Physicians in City at Large. 71; by Irregular Practitioners, 30: by Coroner, 7; by Undertakers, 23.

CAUSES OF DEATH.—Abscess of Perineum 1, Accident 1, Drowning 3. Apoplexy Cerebral, 1, Brain, congestion of, 1, Bronchitis 2, Cholera Infantum, 6, Consumption 8, Convulsions 6, Croup 7, do. Diptheritic 8, Debility 1, Delirium Tremens 2, Diarrhœa 26. Disease of the Brain 1, Diphtheria 6, Dropsy, general, 1, do. of the brain 3, Dysentery 11, Fever, Scarlet, 3, do. Typhoid 9, do. Typhus 1, Gangrene (Hosp.) 1, Inflamation of the Bowels 1, do. Brain 3, do. Brain and Meninges 3, do. Lungs 4, do. Lungs, Typhoid, 4, Infunticide 1, Inanition 1, Marasmus 1, Old Age 5, Premature Birth 1, Pyæmia 1, Suicide 1, Tabes Meseuterica 2—Total deaths from diseases, 411.

The number of de'ths in the first nine months of the present year is 17 less than in the corresponding period of last year, and 1 less than the average for five years.

SANDFORD EASTMAN, M. D., Health Physician.

